# Late Pathomorphological Features of the Endocrine Pancreas in Patients With Type 2 Diabetes Mellitus

**DOI:** 10.7759/cureus.8777

**Published:** 2020-06-22

**Authors:** Irina Tomova, George S Stoyanov, Deyan L Dzhenkov, Lilyana Petkova

**Affiliations:** 1 Medicine, Medical University of Varna, Varna, BGR; 2 General and Clinical Pathology/Forensic Medicine and Deontology, Medical University of Varna, Varna, BGR

**Keywords:** pancreas, amyloid, diabetes, amyloidosis

## Abstract

Introduction

Islet amyloid polypeptide (IAPP) amyloidosis is a pathologic alteration of the pancreas, represented by abnormal accumulation of amylin in the interstitial tissue. Amylin is a neuroendocrine hormone, co-secreted with insulin by beta cells and participating in downstream regulation of postprandial glycemia. This report aims to examine IAPP amyloidosis as a late consequence of poor control of blood glucose levels in patients with type 2 diabetes mellitus (T2DM) who have been referred for autopsy.

Materials and methods

A total of 34 consecutive autopsies performed at the St. Marina University Hospital, Varna, Bulgaria, carried out by a single pathologist were included in the study. Samples from the tail of the pancreas were obtained to evaluate the state of the changes and were analyzed together with the specific organ changes associated with T2DM, as well as the medical documentation of the patients.

Results

Of the 34 autopsies, 10 cases (six females and four males) were included in the study, seven of whom had a medical history of T2D. The average age was 65.7 years (range 50 to 85 years). In all of the cases, morphological features of fibrosis and lipomatosis were present, with one of the patients having signs of pancreatic amyloidosis - Congo red positive deposition of pink, amorphous material in the extracellular matrix.

Conclusion

The described pathological alterations in all of the cases illustrate the progressing impairment of the structure of the pancreas, especially beta cells dysfunction in late stages of T2D, and highlight IAPP amyloidosis as the cause of irreversible damage of the isles of Langerhans and beta cell death.

## Introduction

Islet amyloid polypeptide (IAPP) amyloidosis is a pathologic alteration of the pancreas, represented by abnormal accumulation of amyloid in the interstitial tissue and the isles of Langerhans [1-3]. It is a pathogenic feature of type 2 diabetes mellitus (T2DM) and is associated with the progressing beta cell dysfunction [3-6]. Raising the awareness of this condition and its consequences can contribute to a better understanding of the pathological processes that lay behind it and to advancement in the treatment of T2DM [3,4]. This report aims to examine IAPP amyloidosis as a late result of poor control of blood glucose levels in patients with T2DM who have been referred for autopsy.

## Materials and methods

The study was conducted at the St. Marina University Hospital, Varna and it encompassed a total of 34 consecutive adult autopsies carried out by a single pathologist. Gross observation of all systems was performed and samples from the tail of the pancreas were obtained from each patient. The samples were fixed in 10% buffered formaldehyde and embedded in paraffin. The prepared tissue blocks were cut into 4 mm sections, which were subsequently stained with hematoxylin and eosin (H&E) and Congo red. The findings were further analyzed in conjunction with the specific organ changes associated with T2DM and the medical documentation of the patients. Of all autopsies, 10 cases, six females and four males, were prominent for their pathomorphological changes in the pancreas, which were associated with T2DM and thus, taken into account. Their age range varied from 50 to 83 years (65.7 years on average) and seven of them had a medical history of T2DM. The remaining 24 cases had neither a clinical history nor morphological changes associated with T2DM.

## Results

**Figure 1:**
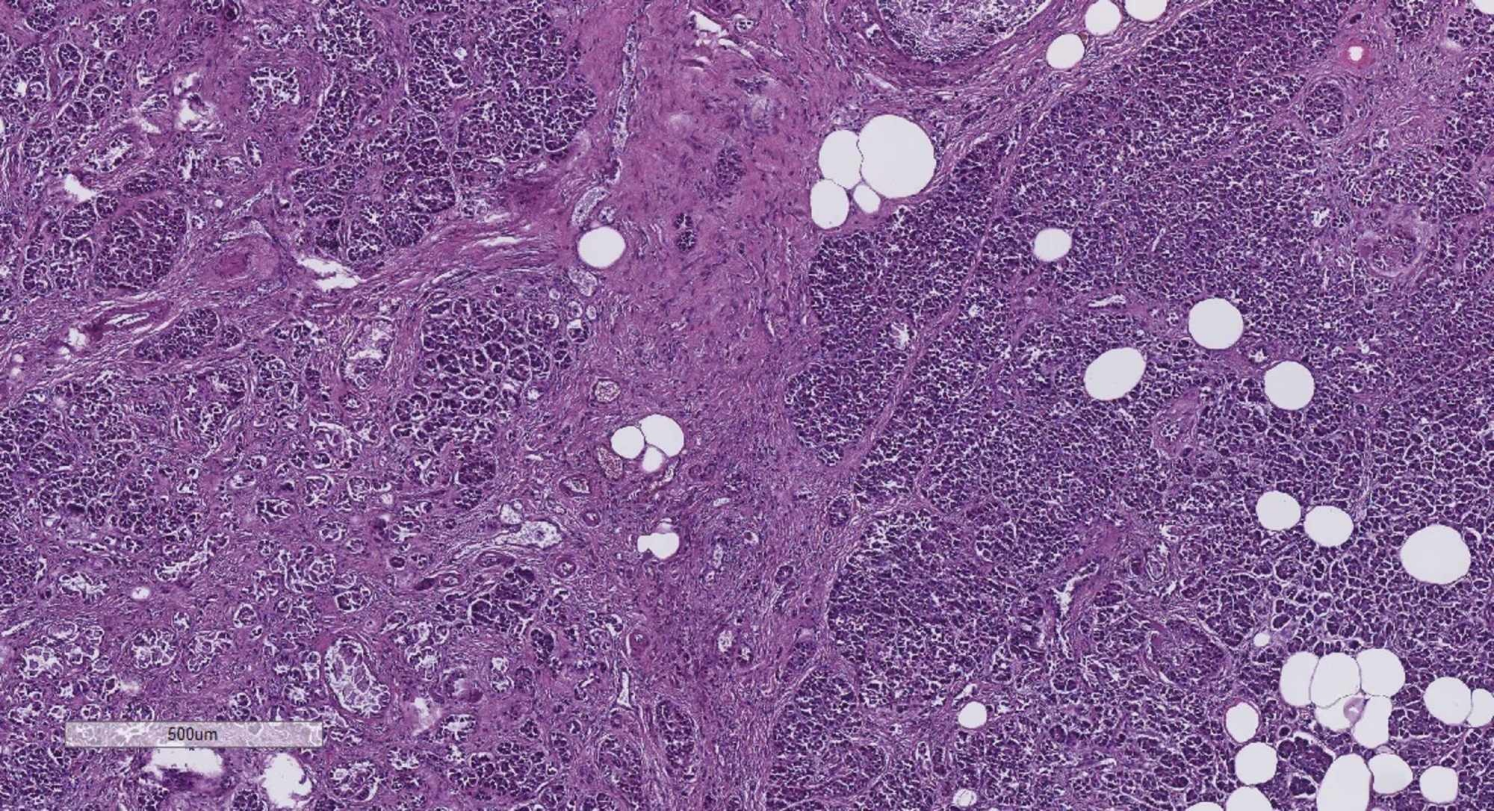
Fibrosis and lipomatosis of the pancreas, H&E stain, original magnification x40 H&E, hematoxylin and eosin

In all 10 cases were present the morphological features of both fibrosis and lipomatosis. On gross examination, the pancreas was hard, shrunken, and the ducts were dilated. Besides, the accumulation of fat tissue could also be noted. All samples were then observed under a light microscope. Fibrosis was demonstrated by solid pink fibrils in the interstitium, which in some cases had started to squeeze through the exocrine acini by pressing and enveloping them (Figure 1). Lipomatosis was represented by big, optically empty cells, filled with lipids (Figure 1). Their distribution was also not limited to the intercellular connective tissue. Rather, single adipocytes or small clusters could be found separating the pancreatic parenchyma. The described changes are the sign that by the progression of fibrosis and lipomatosis, some of the exocrine glands were also affected.

**Figure 2:**
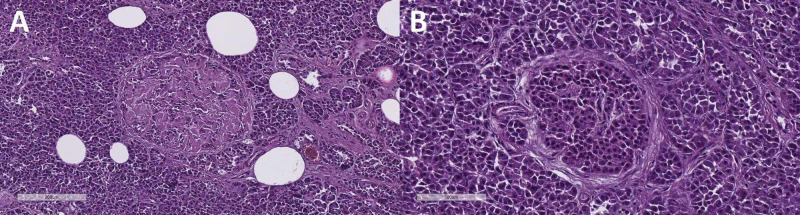
Pancreatic Langerhans isle amyloidosis (A) and healthy age-adjusted control (B). H&E stain, original magnification x100 (A) and x200 (B) H&E, hematoxylin and eosin

One case was prominent for its pink acellular amorphous collections between the cells, which are the morphological substrate of amyloidosis, with the cell mass of the affected isles being significantly reduced (Figure 2). Figure 2B is the normal histological appearance of the isles of Langerhans, from a healthy age-adjusted control. In comparison with Figure 2A, which shows the IAPP amyloidosis and highlights the loss of beta cells.

**Figure 3:**
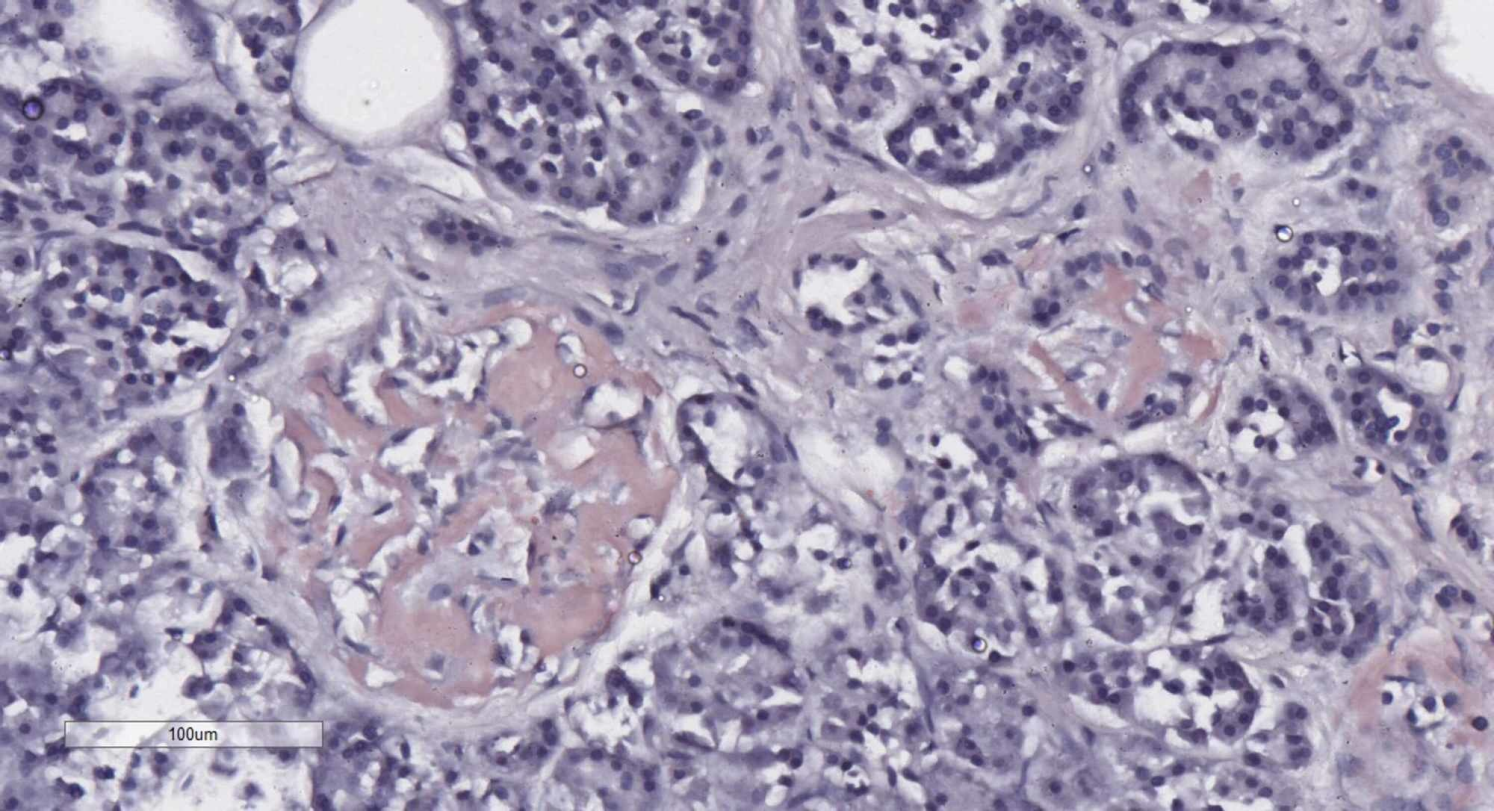
Pancreatic Langerhans isle amyloidosis, Congo red stain, original magnification x200

**Figure 4:**
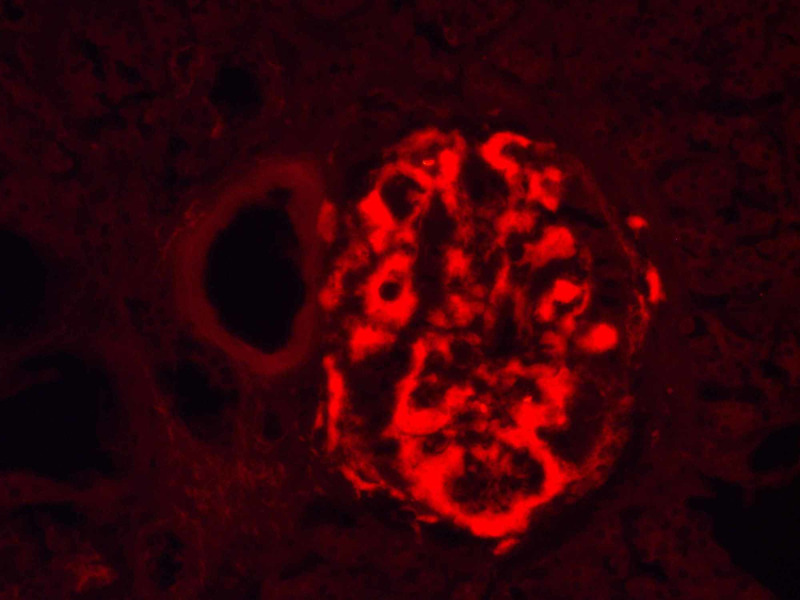
Pancreatic Langerhans isle amyloidosis, Congo red staining observed with fluorescent microscopy, original magnification x200

The affected isles were significantly larger, and their parenchyma was almost totally substituted by the aforementioned material. The accumulations were Congo red positive and their amount was more precisely evaluated by examination of the Congo red-stained sections with fluorescent microscopy (Figures 3, 4).

**Figure 5:**
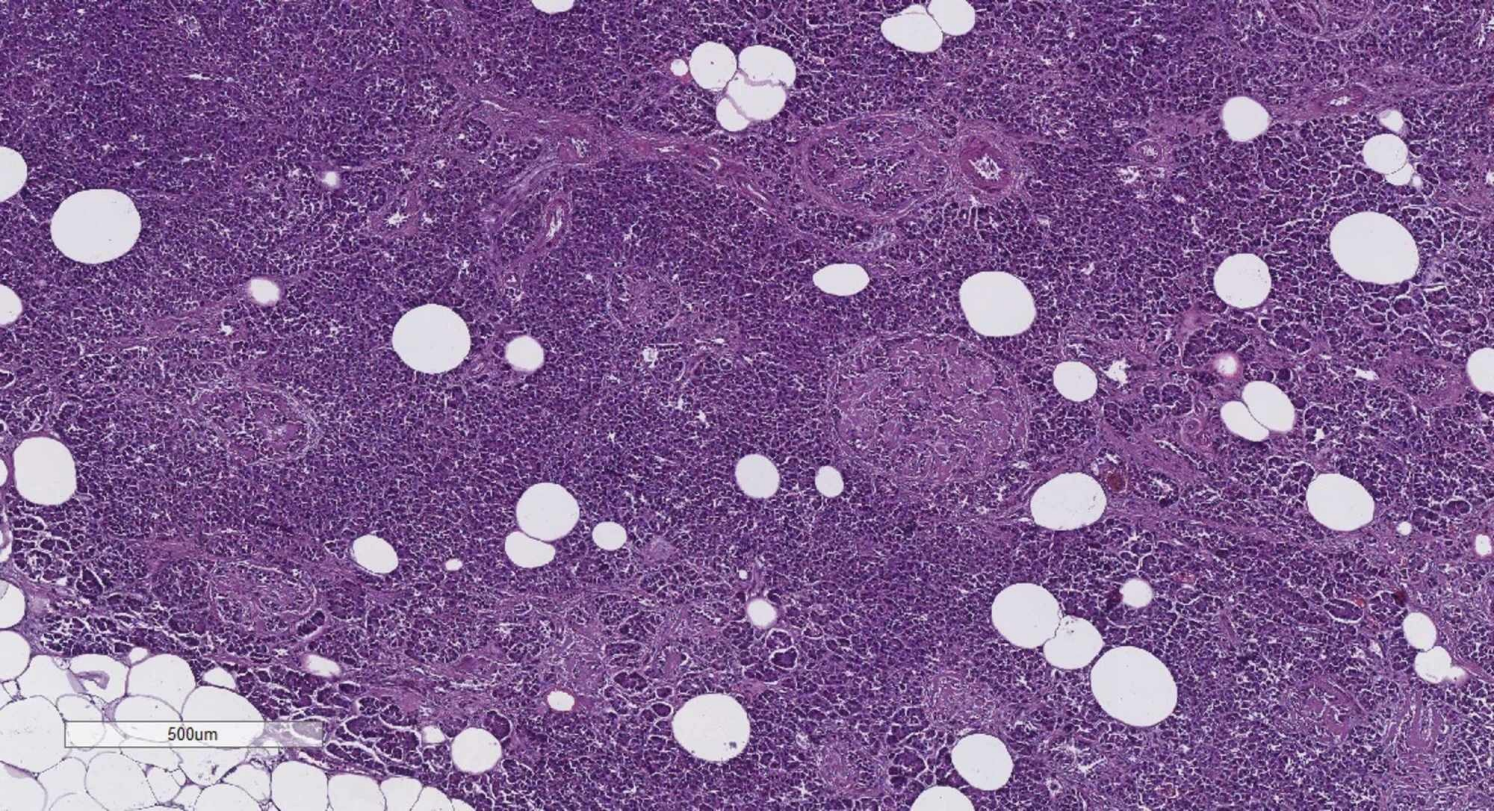
Diffuse pancreatic changes associated with diabetes – fibrosis, lipomatosis and islet amyloidosis, H&E stain, original magnification x40 H&E, hematoxylin and eosin

They showed strong apple-green birefringence on further polarized light microscopy and even distribution through the isle. Furthermore, these changes were not only limited to a small number of isles but also engaged almost all of them (Figure 5). These findings indicate that the process is advanced and the beta cells dysfunction is very prominent.

## Discussion

Amylin, also referred to as IAPP, is a neuroendocrine hormone, co-secreted with insulin by beta cells [1]. It is synthesized as a pro-hormone and undergoes proteolytic processing and participates in downstream regulation of postprandial glycemia [1,2]. Although its role is not yet entirely understood, some studies in humans showed that part of its effects is delayed nutrition delivery from the stomach to the small intestine and inhibited glucagon secretion [3,4]. Moreover, in rats, the hormone targets the central nervous system, particularly the areas responsible for body mass regulation and appetite [5].

Although the precise pathophysiological mechanisms are not yet entirely clear, it has been shown that the development of IAPP coincides with beta cell dysfunction [6-8]. This, in turn, leads to endocrine impairment and the altered chemical environment in the isles associated with T2DM (increased pH, decreased calcium concentration), as they initiate secretion of misfolded monomers [9-11]. These particles have a cytotoxic effect on the beta cells by inducing membrane disruption (formation of abnormal vesicle-like membrane structures) and cell death by either necrosis or apoptosis [12-14]. Their aggregation into less toxic fibrils and plaques constitute the classical representation of IAPP amyloidosis. The deposits result in progressive impairment of the function of beta cells and eventually, reduction of islet cell mass [15]. Furthermore, due to the lost inhibitory effect of amylin, insulin resistance and increased secretion of glucagon are also to be observed. Thus, IAPP amyloidosis should be considered not only as a result, but also a cause of aggravation of T2DM symptoms [11].

IAPP amyloidosis was first observed in 1901 as 'hyaline degeneration of the isles of Langerhans' and a connection between T2DM and this condition was suggested, but it was not until 1987 that the structure of the substance was clarified and further described in other diabetic patients [6,16-18]. Now it is clear that the IAPP amyloidosis is caused by the abnormal accumulation of misfolded amylin and plays a major role in the beta cell destruction [6-8,18]. Our findings are identical to other cases of amyloidosis of the isles in patients with diabetes mellitus and thus consolidate a strong relationship between the two. Although the pathophysiology of the process is yet unclear, certainly, T2DM and the associated alterations in the isles of Langerhans can be regarded as an etiological factor. Therefore, IAPP amyloidosis should be taken into account as a possible complication while treating patients with T2DM, and measures should be taken to avoid its consequences. On the other side, amylin can be regarded not only as a threat but also as a cure; it can be beneficial in the form of pharmaceutical products because of its hypoglycemia characteristics. The amylinomimetics, symlin and pramlintide, exist on the market as antidiabetic drugs and are prescribed as an adjuvant therapy to patients with type 1 and T2DM.

## Conclusions

All of the described pathological alterations illustrate the advanced impairment of both the beta cells and the whole structure of the pancreas in the late stages of T2DM. While fibrosis and lipomatosis affect mostly the interstitial tissue and their effects on the cells are secondary, the IAPP amyloidosis takes place in the isles of Langerhans, damaging the endocrine parenchyma and its secretion directly. Hence, the degeneration of the pancreas is exhaustive, which in turn leads to the progression of the symptoms. The described findings highlight IAPP amyloidosis as one of the main causes of the beta cell dysfunction in the late stages of diabetes mellitus.
